# Genetic diversity and population structure of an extremely endangered species: the world's largest Rhododendron

**DOI:** 10.1093/aobpla/plu082

**Published:** 2014-12-04

**Authors:** Fu Qin Wu, Shi Kang Shen, Xin Jun Zhang, Yue Hua Wang, Wei Bang Sun

**Affiliations:** 1 Present address: School of Life Sciences, Yunnan University, Kunming No. 2, Green Lake North Road, Kunming, Yunnan650091, The People's Republic of China; 2 Kunming Botanical Garden, Kunming Institute of Botany, Chinese Academy of Sciences, Kunming 650201, The People's Republic of China

**Keywords:** AFLP markers, big tree rhododendron, conservation strategies, genetic diversity, *Rhododendron protistum* var. *giganteum*, small population.

## Abstract

Comprehensive studies on the genetic diversity and structure of endangered species are urgently needed to promote effective conservation and management activities. The big tree rhododendron, *Rhododendron protistum* var. *giganteum*, is a highly endangered species with only two known endemic populations in a small area in the southern part of Yunnan Province in China. Unfortunately, limited information is available regarding the population genetics of this species. Therefore, we conducted amplified fragment length polymorphism (AFLP) analysis to characterize the genetic diversity and variation of this species within and between remaining populations. Twelve primer combinations of AFLP produced 447 unambiguous and repetitious bands. Among these bands, 298 (66.67 %) were polymorphic. We found high genetic diversity at the species level (percentage of polymorphic loci = 66.67 %, *h* = 0.240, *I* = 0.358) and low genetic differentiation (*G*_st_ = 0.110) between the two populations. Gene flow between populations (*N*_m_) was relatively high at 4.065. Analysis of molecular variance results revealed that 22 % of the genetic variation was partitioned between populations and 78 % of the genetic variation was within populations. The presence of moderate to high genetic diversity and low genetic differentiation in the two populations can be explained by life history traits, pollen dispersal and high gene flow (*N*_m_ = 4.065). Bayesian structure and principal coordinate analysis revealed that 56 sampled trees were clustered into two groups. Our results suggest that some rare and endangered species are able to maintain high levels of genetic diversity even at small population sizes. These results will assist with the design of conservation and management programmes, such as *in situ* and *ex situ* conservation, seed collection for germplasm conservation and reintroduction.

## Abstract (precis)

This is the first study of the genetic diversity and structure of the big tree rhododendron, *Rhododendron protistum* var. *giganteum*, which is a highly endangered species with only two known endemic populations in a small area in the southern part of Yunnan Province in China. We detected moderate to high genetic diversity at the species level, but low genetic differentiation between the two extant populations. These results suggest that some rare and endangered species are able to maintain high levels of genetic diversity even at small population sizes.

## Introduction

Genetic diversity is one aspect of biological diversity that is extremely important for conservation strategies (Kaljund and Jaaska 2010; Gordon *et al.* 2012). It is well known that preserving the genetic diversity of endangered species can significantly affect their long-term survival and evolution in changing environments (Frankham *et al.* 2002). Therefore, knowledge of the genetic diversity and population structure of endangered plant species is crucial for their conservation and management (Frankham 2003; Gordon *et al.* 2012; Lopes *et al.* 2014). Population size is considered an important factor for maintaining genetic variation. Small populations are more vulnerable than large ones to extinction because of environmental stochasticity, genetic drift and inbreeding. Genetic drift decreases heterozygosity and eventual fixation of alleles, and inbreeding increases homozygosity within populations (Frankham 2005). In general, a drop in population size may cause the decline of genetic diversity by genetic drift and inbreeding. In the longer term, diminished genetic diversity may cause a loss of fitness and evolutionary capacity to adapt to environmental changes (Lande 1993; Kaljund and Jaaska 2010). Therefore, quantifying patterns of genetic variability and diversity within and among different populations is very important for small population species conservation and management planning.

**Figure 1.**
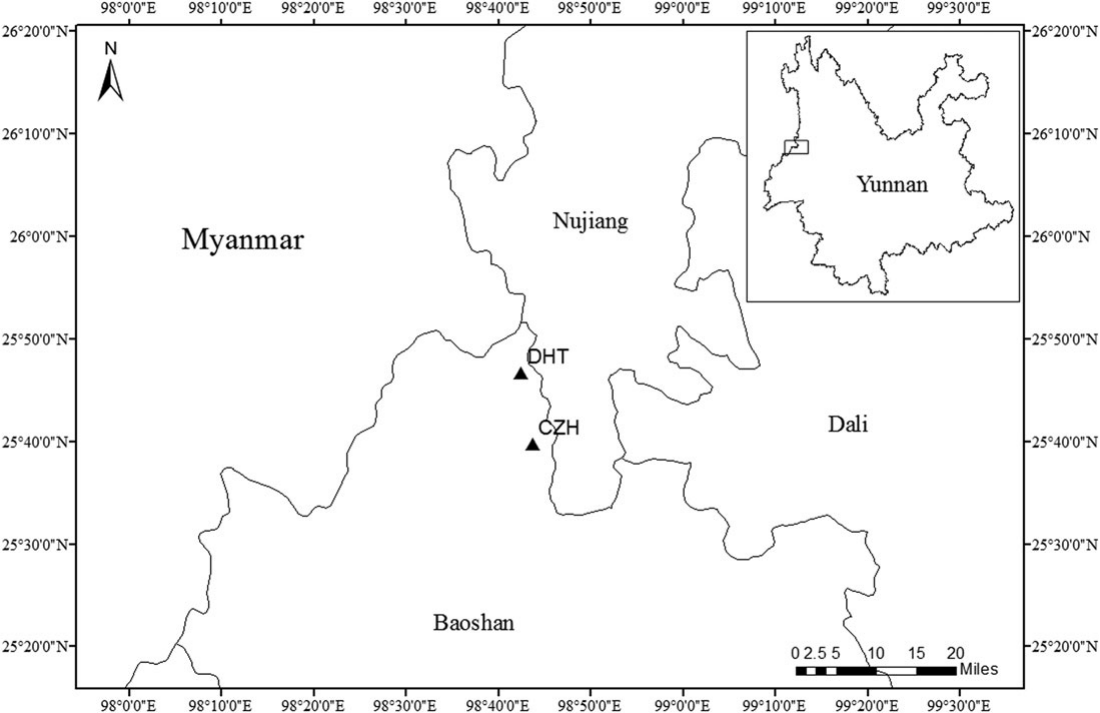
Location of the two populations of big tree rhododendron included in this study. CZH and DHT are population codes.

Big tree rhododendron, *Rhododendron protistum* var. *giganteum*, exhibits a very limited distribution, with only two populations found in the Gaoligong Mountains of northwestern Yunnan Province in China (Fig. 1). In addition, only ∼1500 individual plants have been found (Ma *et al.* 2012). Because of this situation, big tree rhododendron has been included in the Red List of Critically Endangered Species in China (Fu 1992) and protected under the Conservation Programme for Wild Plants with Extremely Small Population in China (2012–15 operational plan) (Ma *et al.* 2013*a*). However, this species is still at risk of extinction because of continued habitat disturbance. Thus, genetic data on big tree rhododendron are urgently needed to inform current and future conservation activities.

In this study, we investigated the genetic diversity and structural patterns of big tree rhododendron both within and between the only known two natural populations. We used amplified fragment length polymorphism (AFLP) markers because this technique has been successfully employed in other studies that evaluated the genetic diversity of other *Rhododendron* species (Escaravage *et al.* 1998; Chappell *et al.* 2008; Erfmeier and Bruelheide 2011; Zhao *et al.* 2012*a*; Li *et al*. 2012*a*). Our study aimed to (i) characterize the level of genetic diversity in big tree rhododendron; (ii) reveal the distribution of genetic variation within and between the two remaining populations; and (iii) discuss possible implications of these population genetic data for management and conservation. We hypothesized that genetic diversity level would be low because of the small size of the remaining populations of big tree rhododendron.

## Methods

### Study species

*Rhododendron* is the largest woody plant genus in the Ericaceae family with at least 1025 species and one of the most common woody plants that are distributed across the northern temperate zone, tropical southeast Asia and northeastern Australia (Chamberlain *et al.* 1996). Approximately 70 % of >500 *Rhododendron* species are endemic in China, and most of these species thrive in the northwestern part of Yunnan province. Thus, northwestern Yunnan has been recognized as one of the diversification and differentiation centres of modern *Rhododendron* (Ma *et al.* 2013*b*). *Rhododendron* species are not only the major composition of alpine and sub-alpine vegetation, but also the world-wide famous woody ornamental plants.

Big tree rhododendron, *R. protistum* var. *giganteum*, was first identified and named by George Forrest in 1919. It belongs to subgenus *Hymenanthes* and subsection *Grandia* (Fang *et al.* 2005). This species is one of the tallest and most ancient rhododendron trees, reaching 30 m in height and 1 m in basal diameter. Big tree rhododendron is an evergreen tree. It is characterized by large, deep and purple-red flowers and oblong-lanceolate to oblong-oblanceolate leaves with continuous and loose indumentums in the abaxial surface. Flowering stage occurs during January to March when flowers abundantly bloom. Fruiting period happens between October and December. Mature seeds are small with thin membranous wings (Fang *et al.* 2005). This plant is a very important germplasm source with high ornamental value.

### Plant materials

According to our previous field investigation, two remaining big tree rhododendron populations are distributed in the Gaoligong Mountain National Nature Reserve, Tengchong County. The Gaoligong Mountains are part of the Hengduan Mountain chain that belongs to a global hotspot for biodiversity. This area is found in the border area between southwestern China and northern Myanmar between 24°40′ and 28°30′N latitude. These mountains cover a total area of 111 000 km^2^. Approximately 3990 species of seed plants with 117 species of *Rhododendron* thrive in this region (Li *et al.* 2000; Ma *et al.* 2013*a*). In November 2012, 56 big tree rhododendron individuals were collected from the two natural populations in which 30 of these samples were collected from the Cizhuhe (CZH) population, whereas 26 samples were collected from the Dahetou (DHT) population. The distance between the collected individual samples was at least 15 m. Fresh young leaves were removed from shoots, dried in silica gel and stored at −20 °C until DNA was extracted. The detailed information regarding locations and population codes of the samples is shown in Fig. 1.

### DNA extraction

Genomic DNA was extracted from dried leaves by using a modified cetyltrimethylammonium bromide protocol (Doyle and Doyle 1987). Total purified DNA was detected by 1.0 % agarose gel electrophoresis and stored at −20 °C until use.

### AFLP fingerprinting

Amplified fragment length polymorphism fingerprinting was performed in accordance with the method described by Vos *et al.* (1995) with minor modifications.

Genomic DNA (150–450 ng) was double-digested using restriction endonucleases EcoRI (5 U) and MseI (5 U) (New England Biolabs, USA) in a total reaction volume of 20 μL for 4 h at 37 °C. This reaction was deactivated at 70 °C for 20 min. The digested DNA (4 µL) was added to 16 µL of the ligation mixture with 50 pmol MseI adaptor, 5 pmol EcoRI adaptor and 2 U T4 DNA ligase (New England Biolabs). This mixture was incubated at 16 °C for 16 h, and then deactivated at 65 °C for 10 min.

The ligated DNA (2 µL) was pre-selected using 33 ng of the primers for MseI and EcoRI adaptors (Table 1) with 5 nmol dNTPs, 10× Taq buffer (2.5 µL with Mg^2+^-free medium) and 1.5 U DNA Taq polymerase (Transgen Biotech, Beijing, China) to yield a total volume of 25 µL. Pre-selective polymerase chain reaction (PCR) amplification profiles are listed as follows: 94 °C for 3 min; 30 cycles of 30 s denaturing at 94 °C, 30 s annealing at 56 °C and 60 s elongation at 72 °C; and holding at 72 °C for 5 min. The pre-selective PCR products were then diluted 10-fold, and the template was used for selective amplifications.

**Table 1.** Polymorphism and primer informativeness of 12 AFLP primer combinations. PPL, percentage of polymorphic loci.

Selective nucl.	Amplification bands	Polymorphism bands	PPL (%)
M-CAA/E-AGC	29	14	48.28
M-CAC/E-AAG	37	27	72.97
M-CAC/E-ACA	44	22	50.00
M-CAC/E-ACC	43	32	74.42
M-CAC/E-AGG	50	28	56.00
M-CAG/E-AAG	31	24	77.42
M-CAG/E-ACC	40	21	52.50
M-CAG/E-ACG	37	29	78.38
M-CAG/E-AGC	36	28	77.78
M-CAT/E-ACG	35	28	80.00
M-CTA/E-ACG	34	27	79.41
M-CTA/E-AGC	31	18	58.06
Total	447	298	–
Mean	37.25	24.83	67.10

An initial screening was performed using two individuals from each area by using 64 primer combinations for selective amplifications. A total of 12 primer combinations (Table 1), which generated clear and abundant bands, were chosen for selective PCR. Selective amplification was performed in a 25-μL reaction volume by using selective primer pairs with 5 nmol dNTPs, 10× Taq buffer (2.5 µL with Mg^2+^-free medium) and 1.5 U DNA Taq polymerase (Transgen Biotech). The selective PCR amplification profile was obtained by denaturation at 94 °C for 30 s, annealing at 65 °C for 30 s, temperature decrease by 0.9 °C/cycle and extension at 72 °C for 1 min for 12 cycles; followed by 94 °C for 30 s, 56 °C for 30 s, 72 °C for 1 min for 23 cycles and 72 °C for 5 min.

Amplified DNA products were mixed with 98 % formamide loading buffer (10 µL), heated at 95 °C for 7 min and immediately cooled in an ice bath for 30 min. The products were resolved by 6 % polyacrylamide gel electrophoresis in 0.5× Tris–borate–EDTA buffer by using a 100-bp DNA ladder marker (Transgen Biotech) for 4 h at 1500 V and then stained with 0.1 % silver nitrate.

### Data analysis

All individuals were manually scored as either ‘1’ or ‘0’ corresponding to the presence or absence of AFLP bands (100–700 bp), respectively, to construct a binary matrix. The binary matrix was edited using GenALEx version 6.4.1 (Peakall and Smouse 2006). The percentage of polymorphic loci (PPL), effective number of alleles (*N*_e_), Nei's genetic diversity (*h*), Shannon's information index (*I*), level of gene flow (*N*_m_), total gene diversity (*H*_t_), variability within populations (*H*_s_) and coefficient of genetic differentiation (*G*_st_) were calculated using POPGENE version 1.32 (Yeh *et al.* 1999) and GenAlEx version 6.4.1 (Peakall and Smouse 2006) with manual corrections.

Analysis of molecular variance (AMOVA) was conducted to calculate the partitioning of genetic variation between and within the two populations by using GenALEx version 6.4.1 (Peakall and Smouse 2006).

We conducted a Bayesian analysis of the population structures by using STRUCTURE version 2.2 (Pritchard *et al.* 2000). A total of 10 independent runs were performed for each set with *K* ranging from 2 to 20, a burn-in of 1 × 10^5^ iterations and 1 × 10^5^ subsequent Markov Chain Monte Carlo steps. The combination of admixture and correlated allele frequency models was also analysed. The second-order rate of change in the log probability of the data with respect to the number of clusters (Δ*K*) was also used to estimate the probable likely number of genetic clusters (Evanno *et al.* 2005). The best-fit number of groupings was evaluated using Δ*K* by STRUCTURE HARVESTER version 0.6.8 (Earl and von Holdt 2012). Furthermore, principal coordinate analysis (PCoA) was also employed to examine the genetic relationships between the detected populations by using GenAlEx version 6.4.1 (Peakall and Smouse 2006).

## Results

### Genetic diversity

Among 64 previously published primer pairs, 12 could amplify well-distributed fragments with good distinction, were highly polymorphic and ranged from 100 to 700 bp. A total of 12 AFLP primers were used in the entire dataset in this study. This dataset generated 447 clear and quantifiable fragments that ranged from 100 to 700 bp in 56 individuals from the two wild populations. Among 447 loci, 298 (66.67 %) were polymorphic. The total number of fragments of each primer combination ranged from 29 (M-CAA/E-AGC) to 50 (M-CAC/E-AGG) with an average of 37.25. Percentage polymorphism varied from 48.28 to 80.00 % with an average of 67.10 % per primer combination (Table 1).

Nei's gene diversity (*h*) and Shannon's information index (*I*), in the combined data matrix of all 12 primers, were 0.240 and 0.358, respectively. The genetic diversities within species (*H*_t_) and within populations (*H*_s_) were 0.238 and 0.212, respectively (Table 2). The genetic differentiation between the populations (*G*_st_) was 0.110. Based on the *G*_st_ value, the level of gene flow (*N*_m_) was estimated as 4.065 (*N*_m_ > 1). These results indicated high gene flow and low differentiation between extant populations.

**Table 2.** Genetic diversity, differentiation parameters of two wild populations of big tree rhododendron. PPL, percentage of polymorphic loci; *h*, Nei's (1973) gene diversity; *I*, Shannon's information index; *H*_t_, total variability; *H*_s_, variability within populations; *G*_st_, coefficient of genetic differentiation; *N*_m_, estimate of gene flow.

Name	PPL (%)	*h*	*I*	*H*_t_	*H*_s_	*G*_st_	*N*_m_
CZH	63.31	0.234	0.346	–	–	–	–
DHT	51.90	0.190	0.281	–	–	–	–
Species level	66.67	0.240	0.358	0.238	0.212	0.110	4.065

At the population level, the CZH population (PPL = 63.31 %, *h* = 0.234, *I* = 0.346) showed a higher genetic diversity level than the DHT population (PPL = 51.90 %, *h* = 0.190, *I* = 0.281; Table 2).

### Genetic structure

Analysis of molecular variance results revealed that 22.00 % of the genetic variation was partitioned between populations and 78.00 % was observed within populations based on AFLP markers (Table 3). These results indicated low genetic variation levels between the two populations.

**Table 3.** Analysis of molecular variance based on AFLP markers for the two populations of big tree rhododendron.

Source of variation	d.f.	Sum of squares	Variation components	Percentage of variation (%)
Among populations	1	355.866	11.318	22.00
Within populations	54	2191.313	40.580	78.00
Total	55	2547.179	51.898	

**Figure 2.**
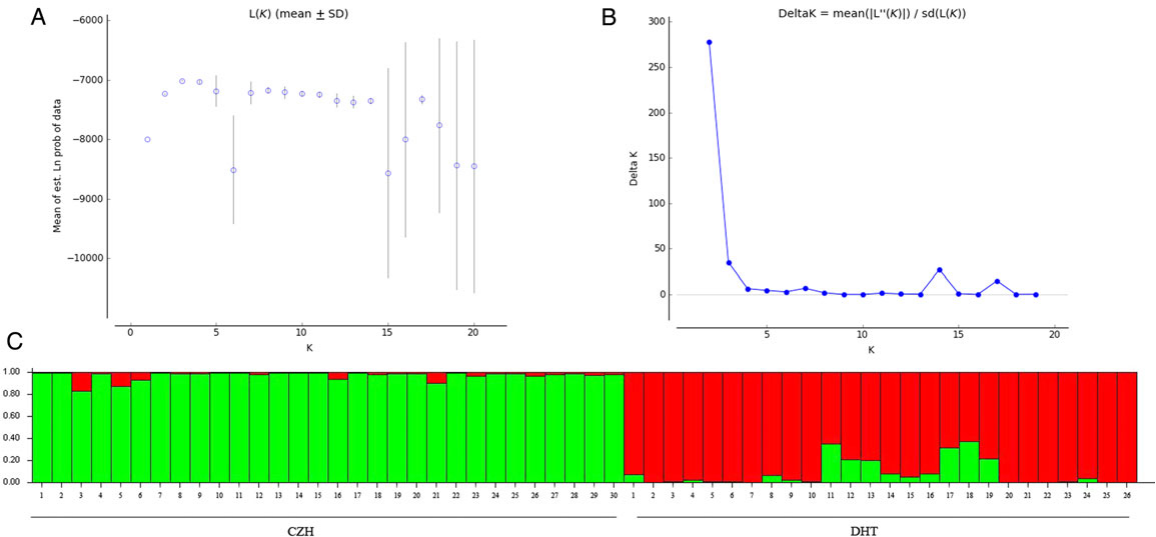
Results of the Bayesian model-based clustering STRUCTURE analysis of 56 individuals of big tree rhododendron. (A) The probability of the data ln *P*(*D*) (±SD) against the number of *K* clusters, and increase of ln *P*(*D*) given *K*, calculated as (Ln*P*(*D*)*k* − Ln*P*(*D*)*k* − 1). (B) Δ*K* values from the mean log-likelihood probabilities from STRUCTURE runs where inferred clusters (*K*) ranged from 1 to 20. (C) Estimated genetic clustering (*K* = 2) obtained with the STRUCTURE program for 56 individuals. Individuals are separated according to the population, and the black vertical line in the bar chart is population identifier.

The genetic structure of big tree rhododendron was analysed on the basis of AFLP markers by using STRUCTURE and PCoA. The STUCTURE analysis based on the Δ*K* method revealed that Δ*K* was 277.7 for *K* = 2 and Δ*K* was <36 for all of the values of *K* (ranging from 3 to 20) (Fig. 2A and B). Therefore, the optimal Δ*K* for *K* = 2 showed that the best-fit model for the sampled 56 individuals of big tree rhododendron revealed two clusters (Fig. 2C). These 56 individuals formed a clear separation between CZH and DHT populations except a few admixed individuals, indicating weak differentiation.

**Figure 3.**
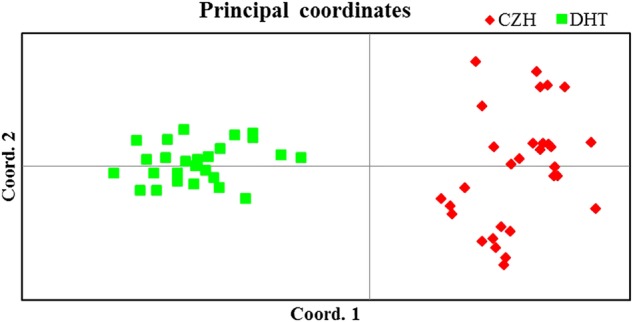
A two-dimensional plot of the PCoA of 56 individuals of big tree rhododendron. The first and second principal coordinates account for 37.74 and 17.89 % of total variation, respectively.

The existence of two groups was also supported by the PCoA (Fig. 3). Two-dimensional PCoA separated the 56 samples into two distinct clusters along the two axes. The F1 axis separated the DHT population, whereas the F2 axis further resolved the CZH population. The first and second principal coordinates accounted for 37.74 and 17.89 % of the total genetic variation, respectively.

## Discussion

### Genetic diversity

The genetic diversity of species in small populations is lower than that in large populations because of genetic drift and inbreeding (Willi *et al.* 2006; Li *et al.* 2012*b*). Therefore, rare and endangered species with narrow geographical distributions likely maintain lower genetic diversity than similar species with widespread geographical distributions (Hamrick and Godt 1989). In the present study, genetic diversity within big tree rhododendron was detected using AFLP markers. Unexpectedly, our study showed that big tree rhododendron showed a higher level of genetic diversity (PPL = 66.67 %, *h* = 0.240, *I* = 0.358) at the species level than other critically endangered tree species such as *Metrosideros bartlettii* (PPL = 44 %) (Drummond *et al.* 2000), *Abies yuanbaoshanensis* (PPL = 50.96 %, *h* = 0.151, *I* = 0.1735) (Wang *et al.* 2003), *Metrosideros boninensis* (PPL = 12.9 %, *h* = 0.024, *I* = 0.039) (Kaneko *et al.* 2008) and *Ostrya rehderiana* (PPL = 29.90 %) (Li *et al.* 2012*b*). In contrast, we found that this rhododendron exhibited a lower level of genetic diversity than reported for *Litsea szemaois* (PPL = 80.8 %) (Ci *et al.* 2008). However, a moderate level of genetic diversity was detected in big tree rhododendron compared with genetic parameters estimated using AFLP markers from other *Rhododendron* species (Chappell *et al.* 2008; Erfmeier and Bruelheide 2011; Zhao *et al.* 2012*a*). The results did not support our hypothesis that big tree rhododendron would exhibit low levels of genetic diversity because of its small populations. Our results generally supported the view that some rare and endangered species can maintain high levels of genetic diversity even at small population sizes (Rossetto *et al.* 1995; Ci *et al.* 2008; Gordon *et al.* 2012; Zhao *et al.* 2012*b*).

The maintenance of high genetic diversity in big tree rhododendron, including its mating system, life form and natural selection, can be explained by several possible reasons. In general, biological traits, reproductive mode and breeding system have often been regarded as important factors that affect genetic diversity levels. Outcrossing species usually have considerably higher levels of genetic diversity than selfing species (Hamrick and Godt 1989; Nybom 2004). Previous studies suggested that the mating system of *Rhododendron* may be predominantly outcrossed because of the need for a pollinator (Hirao *et al.* 2006; Ono *et al.* 2008; Hirao 2010). In a field survey, big tree rhododendron is pollinated by insect vectors and birds (S. K. Shen, unpubl. data), which largely promote outcrossing. The seeds of big tree rhododendron are small with wing-like structures that allow them to be dispersed by the wind. Moreover, tree species, even though their populations are declining, usually maintain higher levels of genetic polymorphisms than short-lived herbaceous species (Hamrick and Godt 1989; Nybom 2004). The big tree rhododendron lives for decades to centuries, and this characteristic is highly advantageous to retain genetic variation. The big tree rhododendron is only distributed in two neighbouring populations with small ranges. The two remaining populations may exhibit high genetic diversity derived from the ancestral population; this result is similar to that in a previous study focusing on another critically endangered plant, namely, *Tricyrtis ishiiana* (Setoguchi *et al.* 2011).

High genetic variation enables species to adapt to changing environments (Zhao *et al.* 2012*b*). The presence of moderate to high genetic diversity in the two populations of big tree rhododendron indicated that the current endangered status of this species is not caused by genetic factors (e.g. genetic diversity decline, genetic drift and inbreeding); this result is similar to that in another endangered species, namely, *Tupistra pingbianensis* (Qiao *et al.* 2010). The main threat to this plant species may be habitat specialization. Although we did not conduct a detailed habitat survey, the limited distribution and few seedlings found in the wild may partly support this hypothesis. However, the factors that lead to the sustainable declining of this population should be further elucidated.

### Genetic structure

Genetic structure is affected by several factors, such as breeding systems, genetic drift, population size, seed dispersal, gene flow, evolutionary history and natural selection (Hamrick and Godt 1990). Our analyses of genetic structure showed that the 56 individuals formed a clear separation between CZH and DHT populations except a few admixed individuals; this result indicated weak differentiation (Fig. 2). This conclusion is also supported by the PCoA (Fig. 3). Analysis of molecular variance analysis of two populations showed that 22 % of the genetic variation occurred between CZH and DHT populations, whereas 78 % of the genetic variation occurred within these populations (Table 3). The coefficients of genetic differentiation (*G*_st_) and gene flow (*N*_m_) between the two extant populations were 0.110 and 4.065, respectively. Long distance dispersal of pollen and/or seeds results in low genetic differentiation and high gene flow between populations of the same species (Yao *et al.* 2007; Zhao *et al.* 2012*b*). Previous studies found that the seeds of *Rhododendron* species are frequently dispersed by the wind, and the distance of seed dispersal approximately ranges from 30 to 80 m (Ng and Corlett 2000). However, *Rhododendron* pollen can also be transmitted by insect vectors (bees) and birds (Hirao *et al.* 2006; Ono *et al.* 2008; Hirao 2010). Moreover, these pollens can commonly be moved at a distance ranging from 3 to 10 km (Ng and Corlett 2000). The geographical distance between the two extant populations of big tree rhododendron is ∼8 km. Thus, it is reasonable to assume that the frequent and continual gene flow between the two populations of big tree rhododendron occurs by pollen dispersal.

## Conservation implications

Our study of the genetic structure of big tree rhododendron has important implications for the conservation and management of this narrowly distributed and extremely rare species. Undoubtedly, *in situ* conservation is considered as the most effective method to conserve endangered species (Shen *et al.* 2009). The presence of high genetic diversity in big tree rhododendron indicates that the major factors that threaten the persistence of its population are ecological factors (e.g. habitat specialization) rather than genetic. Considering its habitat specialization and extremely limited distribution, we suggest that management policies should be improved to maintain the appropriate effective population size of big tree rhododendron and to protect its natural habitats. Furthermore, previous studies proposed that mature individuals in populations should be conserved to protect reproductive fitness and evolutionary potential of the species (Cruse-Sanders *et al.* 2005). For instance, adult big tree rhododendrons are critical resources, not only to maintain current genetic diversity but also to provide provenance for its future recovery. Thus, protecting adult trees should be the priority in conservation to ensure ongoing recruitment. In addition, *ex situ* conservation is important to support the recovery of wild populations. The two existing populations have unique genotypes, as detected in the Bayesian clustering analysis. With these findings, we recommend that seeds be collected for germplasm storage and *ex situ* conservation of both the CZH and DHT populations.

## Conclusions

Population genetic diversity and structure of extremely small populations of big tree rhododendron were examined in this study using AFLP markers, and we detected moderate to high genetic diversity at the species level, but low genetic differentiation between the two extant populations. These results suggest that some rare and endangered species are able to maintain high levels of genetic diversity even at small population sizes. Our hope is that these results will help design species conservation and management programmes, such as *in situ* and *ex situ* conservation, seed collection for germplasm conservation and reintroduction.

## Sources of Funding

This study was financially supported by grants 31360155 and U1302262 from the National Science Foundation of China and grant 2011FB001 from the Application foundation projects of Yunnan Province and Graduate Science Innovation projects of Yunnan University (ynuy201343).

## Contributions by the Authors

F.Q.W., S.K.S., Y.H.W., W.B.S. initiated and designed the research; S.K.S. and W.B.S. obtained funding for the study; S.K.S., F.Q.W. and X.J.Z. collected materials and performed the experiments, F.Q.W. and S.K.S. analysed the data and wrote the paper. All authors read, edited and agreed to submit the manuscript.

## Conflict of Interest Statement

None declared.
